# Supplementary material for: Differences in sensory nerve block between levobupivacaine and bupivacaine at low concentrations in humans and animals
Source: PLoS One. 2025 Feb 10;20(2):e0306591. doi: 10.1371/journal.pone.0306591 (PMC11809910; doi:10.1371/journal.pone.0306591)
Supplement: S1 Table — (DOCX) [file pone.0306591.s001.docx]

**Supplementary Table S1.** Carryover effect and period effect

|  | **Sequence A (n = 9)** | | **Sequence B (n = 9)** | | ***p* value for Carryover effect** | ***p* value for Period effect** |
| --- | --- | --- | --- | --- | --- | --- |
| **Variable** | **1st period**  **(Bup)** | **2nd period**  **(Lev)** | **1st period**  **(Lev)** | **2nd period**  **(Bup)** |  |  |
| At baseline  TDT (g)  MPT (g)  TPT (°C) | 0.75 ± 0.31  11.11 ± 2.20  45.24 ± 0.48 | 0.62 ± 0.16  11.11 ± 2.20  45.10 ± 0.57 | 0.69 ± 0.20  10.56 ± 1.67  45.06 ± 0.80 | 0.53 ± 0.10  11.11 ± 2.20  44.75 ± 0.88 | 0.1371  0.7466  0.2006 | 0.1072  0.5868  0.4112 |
| At 15 min after Bup or Lev  TDT (g)  MPT (g)  TPT (°C) | 29.00 ± 31.42  202.22 ± 80.28  46.79 ± 0.33 | 14.78 ± 32.02  237.78 ± 77.75  46.80 ± 0.32 | 5.27 ± 4.77  206.67 ± 93.81  46.51 ± 0.63 | 23.44 ± 21.65  180.00 ± 97.98  46.73 ± 0.36 | 0.3642  0.3131  0.1668 | 0.8212  0.8931  0.4876 |

Data are expressed as means ± standard deviation. Abbreviations: Bup, bupivacaine; Lev, levobupivacaine; TDT, tactile detection threshold; MPT, mechanical pain threshold; TPT, thermal pain threshold.
