# Supplementary material for: Differences in sensory nerve block between levobupivacaine and bupivacaine at low concentrations in humans and animals
Source: PLoS One. 2025 Feb 10;20(2):e0306591. doi: 10.1371/journal.pone.0306591 (PMC11809910; doi:10.1371/journal.pone.0306591)
Supplement: S2 Table — (DOCX) [file pone.0306591.s002.docx]

**Supplementary Table S2.** Experiment 1 (human study)

Tactile detective thresholds (g)

|  | Concentration (%) | baseline | 5 | 15 | 30 | 45 | 60  (min) |
| --- | --- | --- | --- | --- | --- | --- | --- |
| Levobupivacaine | 0.125 | 1 | 8 | 10 | 8 | 6 | 4 |
|  | 0.125 | 0.6 | 60 | 100 | 60 | 60 | 10 |
|  | 0.125 | 0.4 | 4 | 6 | 4 | 2 | 2 |
|  | 0.125 | 0.6 | 8 | 4 | 2 | 2 | 1 |
|  | 0.125 | 0.6 | 6 | 4 | 4 | 2 | 1 |
|  | 0.125 | 0.4 | 15 | 15 | 4 | 1 | 1 |
| Bupivacaine | 0.125 | 0.4 | 60 | 8 | 6 | 8 | 6 |
|  | 0.125 | 0.6 | 60 | 100 | 8 | 8 | 1.4 |
|  | 0.125 | 0.4 | 60 | 15 | 8 | 6 | 6 |
|  | 0.125 | 0.6 | 8 | 60 | 10 | 10 | 10 |
|  | 0.125 | 0.6 | 100 | 10 | 8 | 8 | 6 |
|  | 0.125 | 0.4 | 15 | 8 | 2 | 2 | 1 |
|  | concentration | baseline | 5 | 15 | 30 | 45 | 60 |
| Levobupivacaine | 0.0625 | 0.6 | 2 | 4 | 2 | 1.4 | 1 |
|  | 0.0625 | 0.6 | 15 | 8 | 8 | 2 | 1 |
|  | 0.0625 | 0.6 | 26 | 4 | 1 | 1.4 | 0.6 |
|  | 0.0625 | 1 | 8 | 8 | 2 | 2 | 1 |
|  | 0.0625 | 0.6 | 2 | 4 | 2 | 1.4 | 1 |
|  | 0.0625 | 0.8 | 8 | 4 | 2 | 1 | 1 |
| Bupivacaine | 0.0625 | 0.6 | 60 | 26 | 2 | 2 | 1 |
|  | 0.0625 | 0.6 | 60 | 26 | 15 | 10 | 8 |
|  | 0.0625 | 0.6 | 100 | 60 | 8 | 6 | 2 |
|  | 0.0625 | 0.4 | 26 | 15 | 4 | 4 | 4 |
|  | 0.0625 | 0.6 | 6 | 4 | 4 | 4 | 2 |
|  | 0.0625 | 0.6 | 60 | 15 | 4 | 2 | 1 |
|  | concentration | baseline | 5 | 15 | 30 | 45 | 60 |
| Levobupivacaine | 0.025 | 1 | 2 | 2 | 1.4 | 1 | 1 |
|  | 0.025 | 0.6 | 2 | 1 | 1 | 1 | 1 |
|  | 0.025 | 0.6 | 1 | 1 | 1 | 0.6 | 1 |
|  | 0.025 | 0.6 | 2 | 2 | 1.4 | 1 | 0.6 |
|  | 0.025 | 0.6 | 2 | 1.4 | 0.6 | 0.6 | 0.6 |
|  | 0.025 | 0.6 | 1.4 | 2 | 0.6 | 0.6 | 0.6 |
| Bupivacaine | 0.025 | 1 | 6 | 8 | 10 | 8 | 4 |
|  | 0.025 | 1.4 | 10 | 15 | 10 | 8 | 6 |
|  | 0.025 | 1 | 4 | 8 | 4 | 1 | 1 |
|  | 0.025 | 0.6 | 26 | 8 | 4 | 1 | 0.6 |
|  | 0.025 | 0.6 | 60 | 60 | 4 | 1 | 0.6 |
|  | 0.025 | 0.6 | 10 | 26 | 8 | 6 | 1.4 |

(g)

Mechanical pain thresholds (g)

|  | Concentration  (%) | baseline | 5 | 15 | 30 | 45 | 60  (min) |
| --- | --- | --- | --- | --- | --- | --- | --- |
| Levobupivacaine | 0.125 | 15 | 180 | 300 | 60 | 100 | 15 |
|  | 0.125 | 10 | 300 | 300 | 300 | 300 | 300 |
|  | 0.125 | 15 | 180 | 300 | 300 | 180 | 100 |
|  | 0.125 | 10 | 100 | 180 | 100 | 60 | 10 |
|  | 0.125 | 10 | 100 | 180 | 100 | 26 | 10 |
|  | 0.125 | 10 | 300 | 300 | 180 | 60 | 15 |
| Bupivacaine | 0.125 | 10 | 300 | 100 | 60 | 100 | 100 |
|  | 0.125 | 10 | 300 | 300 | 300 | 100 | 26 |
|  | 0.125 | 10 | 300 | 100 | 100 | 100 | 100 |
|  | 0.125 | 15 | 300 | 300 | 180 | 100 | 60 |
|  | 0.125 | 15 | 300 | 100 | 26 | 26 | 15 |
|  | 0.125 | 10 | 300 | 180 | 60 | 26 | 15 |
|  | concentration | baseline | 5 | 15 | 30 | 45 | 60 |
| Levobupivacaine | 0.0625 | 10 | 180 | 300 | 180 | 100 | 26 |
|  | 0.0625 | 15 | 300 | 300 | 300 | 100 | 60 |
|  | 0.0625 | 10 | 300 | 300 | 100 | 60 | 10 |
|  | 0.0625 | 10 | 300 | 180 | 180 | 100 | 26 |
|  | 0.0625 | 10 | 180 | 300 | 180 | 100 | 26 |
|  | 0.0625 | 10 | 300 | 300 | 180 | 60 | 15 |
| Bupivacaine | 0.0625 | 10 | 300 | 180 | 26 | 15 | 15 |
|  | 0.0625 | 10 | 300 | 300 | 300 | 300 | 300 |
|  | 0.0625 | 10 | 300 | 180 | 60 | 15 | 15 |
|  | 0.0625 | 10 | 100 | 60 | 26 | 26 | 15 |
|  | 0.0625 | 15 | 100 | 100 | 60 | 60 | 26 |
|  | 0.0625 | 10 | 300 | 180 | 60 | 26 | 15 |
|  | concentration | baseline | 5 | 15 | 30 | 45 | 60 |
| Levobupivacaine | 0.025 | 10 | 180 | 180 | 26 | 10 | 10 |
|  | 0.025 | 10 | 180 | 100 | 180 | 60 | 15 |
|  | 0.025 | 10 | 26 | 180 | 100 | 100 | 15 |
|  | 0.025 | 10 | 300 | 100 | 100 | 60 | 10 |
|  | 0.025 | 10 | 180 | 100 | 10 | 10 | 10 |
|  | 0.025 | 10 | 100 | 100 | 10 | 10 | 10 |
| Bupivacaine | 0.025 | 10 | 26 | 180 | 180 | 100 | 15 |
|  | 0.025 | 15 | 300 | 180 | 100 | 60 | 26 |
|  | 0.025 | 10 | 60 | 300 | 100 | 10 | 10 |
|  | 0.025 | 10 | 300 | 100 | 15 | 10 | 10 |
|  | 0.025 | 10 | 300 | 300 | 26 | 15 | 10 |
|  | 0.025 | 10 | 100 | 100 | 100 | 60 | 26 |

(g)

Thermal pain thresholds (°C)

|  | Concentration  (%) | baseline | 5 | 15 | 30 | 45 | 60  (min) |
| --- | --- | --- | --- | --- | --- | --- | --- |
| Levobupivacaine | 0.125 | 45.3 | 47 | 47 | 47 | 47 | 46.2 |
|  | 0.125 | 44.3 | 47 | 47 | 47 | 47 | 46.2 |
|  | 0.125 | 45.3 | 47 | 46.8 | 46.4 | 46.3 | 46.2 |
|  | 0.125 | 45.2 | 46.4 | 46.8 | 45.7 | 45.8 | 45.4 |
|  | 0.125 | 45.4 | 46.4 | 46.7 | 45.9 | 46.1 | 45.6 |
|  | 0.125 | 45.9 | 47 | 47 | 47 | 47 | 46.2 |
| Bupivacaine | 0.125 | 44.3 | 47 | 46.6 | 45.8 | 44.8 | 43.8 |
|  | 0.125 | 45.3 | 47 | 47 | 47 | 47 | 45.6 |
|  | 0.125 | 45.3 | 47 | 46.6 | 45.4 | 44.6 | 43.8 |
|  | 0.125 | 45.3 | 45.5 | 46.8 | 46.6 | 45.3 | 45.1 |
|  | 0.125 | 45.5 | 47 | 47 | 46.3 | 46.3 | 46.1 |
|  | 0.125 | 44.2 | 47 | 47 | 46.5 | 46.1 | 45.9 |
|  | concentration | baseline | 5 | 15 | 30 | 45 | 60 |
| Levobupivacaine | 0.0625 | 45.3 | 46.3 | 46.9 | 46.3 | 45.6 | 45.3 |
|  | 0.0625 | 45.6 | 47 | 47 | 47 | 46.2 | 46 |
|  | 0.0625 | 45.8 | 47 | 47 | 47 | 46.2 | 45.3 |
|  | 0.0625 | 45.8 | 46.8 | 46.8 | 46.2 | 46 | 45.6 |
|  | 0.0625 | 45.3 | 46.3 | 46.9 | 46.3 | 45.6 | 45.3 |
|  | 0.0625 | 45.4 | 46.8 | 46.4 | 46.2 | 45.8 | 45.6 |
| Bupivacaine | 0.0625 | 45 | 46.4 | 46 | 45.4 | 44.2 | 44 |
|  | 0.0625 | 45.5 | 47 | 47 | 47 | 46.1 | 45.8 |
|  | 0.0625 | 45.8 | 47 | 46.8 | 47 | 46.2 | 46 |
|  | 0.0625 | 45.1 | 47 | 47 | 47 | 46.8 | 46.3 |
|  | 0.0625 | 46.1 | 47 | 46.9 | 46.9 | 47 | 47 |
|  | 0.0625 | 45.2 | 46.6 | 46 | 45.2 | 44 | 44 |
|  | concentration | baseline | 5 | 15 | 30 | 45 | 60 |
| Levobupivacaine | 0.025 | 44.9 | 46.9 | 47 | 45.4 | 45.1 | 44.9 |
|  | 0.025 | 44.1 | 46.1 | 46 | 43.8 | 45.1 | 43.2 |
|  | 0.025 | 45 | 46 | 47 | 47 | 47 | 46.3 |
|  | 0.025 | 43.5 | 47 | 45.1 | 45.2 | 43.2 | 43 |
|  | 0.025 | 44 | 46.4 | 46.2 | 44 | 44 | 44 |
|  | 0.025 | 45.3 | 47 | 46.2 | 45.8 | 45.3 | 45.3 |
| Bupivacaine | 0.025 | 45 | 44 | 47 | 47 | 45.8 | 44 |
|  | 0.025 | 45.8 | 47 | 47 | 46.8 | 46.3 | 46 |
|  | 0.025 | 45 | 46.5 | 46.7 | 44.9 | 44.9 | 45 |
|  | 0.025 | 43.9 | 46.1 | 46.3 | 44.4 | 44.4 | 44.2 |
|  | 0.025 | 44.5 | 47 | 47 | 45.6 | 44.5 | 44.5 |
|  | 0.025 | 43.2 | 45.9 | 47 | 46 | 45.2 | 45.8 |

(℃)
