# Supplementary material for: Differences in sensory nerve block between levobupivacaine and bupivacaine at low concentrations in humans and animals
Source: PLoS One. 2025 Feb 10;20(2):e0306591. doi: 10.1371/journal.pone.0306591 (PMC11809910; doi:10.1371/journal.pone.0306591)
Supplement: S3 Table — (DOCX) [file pone.0306591.s003.docx]

**Supplementary Table S3.** Experiment 2 (animal study)

% change of discharge frequency of WDR neuron

| **Brush** | baseline | 5 | 10 | 20 | 30 | 60 | 90 (min) |
| --- | --- | --- | --- | --- | --- | --- | --- |
| Levobupivacaine (0.05%) | 0 | -22.7273 | -18.1818 | -50 | -40.9091 | -22.7273 | 18.18182 |
|  | 0 | -57.1429 | -61.9047 | -52.381 | 4.761905 | 4.761905 | 95.2381 |
|  | 0 | -37.931 | -27.5862 | -51.7241 | -62.069 | -75.8621 | -62.069 |
|  | 0 | 5.714286 | 20 | -22.8571 | -2.85714 | -2.85714 | 11.42857 |
|  | 0 | -33.3333 | -33.3333 | -8.33333 | -25 | 16.66667 | 91.66667 |
| Bupivacaine (0.05%) | 0 | -65.2174 | -82.6086 | -95.6522 | -95.6522 | -69.5652 | -43.4783 |
|  | 0 | -57.1429 | -35.7142 | -78.5714 | -78.5714 | -85.7143 | -35.7143 |
|  | 0 | -81.5789 | -84.2105 | -92.1053 | -86.8421 | -86.8421 | 36.84211 |
|  | 0 | -43.5897 | -71.7948 | -97.4359 | -97.4359 | -97.4359 | -89.7436 |
|  | 0 | -88.2353 | -94.1176 | -88.2353 | -94.1176 | -94.1176 | -76.4706 |
| NS | 0 | -10 | 25 | 0 | 10 | 55 | 40 |
|  | 0 | -8.33333 | -5.55556 | 13.88889 | 11.11111 | 11.11111 | 16.66667 |
|  | 0 | -18.3673 | -2.04082 | -4.08163 | -4.08163 | -8.16327 | 8.163265 |

| **vF 4** | baseline | 5 | 10 | 20 | 30 | 60 | 90 (min) |
| --- | --- | --- | --- | --- | --- | --- | --- |
| Levobupivacaine (0.05%) | 0 | -40 | 40 | 20 | 40 | 80 | 100 |
|  | 0 | -76.4706 | -94.1176 | -94.1176 | -41.1765 | -17.6471 | -29.4118 |
|  | 0 | 0 | -66.6667 | -53.3333 | -66.6667 | -40 | -66.6667 |
|  | 0 | 5.882353 | -5.88235 | -41.1765 | -29.4118 | 23.52941 | 23.52941 |
|  | 0 | -50 | -62.5 | -50 | -62.5 | -62.5 | 0 |
| Bupivacaine (0.05%) | 0 | -90 | -100 | -100 | -95 | -90 | -75 |
|  | 0 | -11.1111 | -33.3333 | -88.8889 | -88.8889 | -88.8889 | -44.4444 |
|  | 0 | -69.2308 | -84.6153 | -100 | -92.3077 | -76.9231 | 7.692308 |
|  | 0 | -10 | -90 | -90 | -100 | -100 | -70 |
|  | 0 | -85.7143 | -100 | -100 | -100 | -100 | -85.7143 |
| NS | 0 | -27.2727 | 6.060606 | -24.2424 | 12.12121 | 27.27273 | -18.1818 |
|  | 0 | 18.66667 | 6.666667 | 1.333333 | 0 | 1.333333 | 13.33333 |
|  | 0 | 2.307692 | 12.30769 | 14.61538 | 3.076923 | 10 | 13.84615 |

| **Pinch** | baseline | 5 | 10 | 20 | 30 | 60 | 90 (min) |
| --- | --- | --- | --- | --- | --- | --- | --- |
| Levobupivacaine (0.05%) | 0 | -66.6667 | -87.5 | -91.6667 | -87.5 | -70.8333 | -37.5 |
|  | 0 | -76.1905 | -80.9523 | -90.4762 | -85.7143 | 38.09524 | 100 |
|  | 0 | -69.2308 | -84.6153 | -92.3077 | -92.3077 | -88.4615 | -50 |
|  | 0 | -87.619 | -92.3809 | -88.5714 | -90.4762 | -92.381 | -60.9524 |
|  | 0 | -64.7059 | -88.2352 | -76.4706 | -76.4706 | -64.7059 | 17.64706 |
| Bupivacaine (0.05%) | 0 | -81.8182 | -100 | -100 | -96.9697 | -54.5455 | 100 |
|  | 0 | 0 | -52.3809 | -85.7143 | -85.7143 | -71.4286 | -4.7619 |
|  | 0 | -82.5 | -85 | -82.5 | -87.5 | -52.5 | 5 |
|  | 0 | -68.8889 | -84.4444 | -84.4444 | -91.1111 | -91.1111 | -20 |
|  | 0 | -83.3333 | -87.5 | -91.6667 | -91.6667 | -87.5 | -12.5 |
| NS | 0 | -20.4225 | -21.1268 | -47.8873 | -35.2113 | -19.7183 | -28.169 |
|  | 0 | 9.285714 | 10 | 7.142857 | 6.428571 | 5 | 7.857143 |
|  | 0 | 4.621849 | -3.36134 | -11.5546 | 3.781513 | -1.68067 | -5.88235 |

| **vF 10** | baseline | 5 | 10 | 20 | 30 | 60 | 90 (min) |
| --- | --- | --- | --- | --- | --- | --- | --- |
| Levobupivacaine (0.05%) | 0 | -68.4211 | -94.7368 | -68.4211 | -89.4737 | -68.4211 | -36.8421 |
|  | 0 | -92 | -96 | -96 | -76 | -48 | 24 |
|  | 0 | -87.5 | -93.75 | -81.25 | -93.75 | -93.75 | -75 |
|  | 0 | -61.5385 | -84.6153 | -84.6154 | -84.6154 | -65.3846 | -7.69231 |
|  | 0 | -71.4286 | -85.7142 | -85.7143 | -71.4286 | -42.8571 | 28.57143 |
| Bupivacaine (0.05%) | 0 | -83.3333 | -100 | -91.6667 | -91.6667 | -66.6667 | -41.6667 |
|  | 0 | -35.7143 | -35.7142 | -92.8571 | -92.8571 | -92.8571 | -57.1429 |
|  | 0 | -75 | -83.3333 | -75 | -75 | -33.3333 | 141.6667 |
|  | 0 | -75 | -83.3333 | -91.6667 | -95.8333 | -91.6667 | -54.1667 |
|  | 0 | -90 | -90 | -100 | -100 | -90 | 10 |
| NS | 0 | -12 | -38.6667 | -32 | -26.6667 | -32 | -34.6667 |
|  | 0 | 0 | 0 | -15 | -5 | -11 | -13 |
|  | 0 | -20.3774 | -1.50943 | -9.43396 | -3.77358 | -0.9434 | 0 |

% change of discharge frequency of LT neuron

| **Brush** | baseline | 5 | 10 | 20 | 30 | 60 | 90 (min) |
| --- | --- | --- | --- | --- | --- | --- | --- |
| Levobupivacaine (0.05%) | 0 | -25.7895 | 0 | -38.5965 | 12.63158 | 11.57895 | 15.78947 |
|  | 0 | -18.8755 | -16.4659 | -0.80321 | 20.48193 | 1.606426 | 4.417671 |
|  | 0 | -58.9071 | -50.1639 | -23.9344 | -16.0656 | -5.13661 | 4.043716 |
|  | 0 | -14 | -15.7143 | 2.857143 | 9.714286 | 12.57143 | 2.857143 |
|  | 0 | 2.380952 | -8.33333 | 0 | -1.78571 | 14.28571 | -10.7143 |
| Bupivacaine (0.05%) | 0 | -63.1769 | -68.231 | -70.7581 | -48.7365 | -19.4946 | 3.971119 |
|  | 0 | -65.2299 | -72.7011 | -64.3678 | -29.023 | -33.3333 | -12.3563 |
|  | 0 | -3.78788 | -1.89394 | -63.1818 | -41.7045 | -44.4318 | -25 |
|  | 0 | -49.4585 | -48.0144 | -22.3827 | -12.2744 | -7.22022 | -1.08303 |
|  | 0 | -37.5546 | -55.4585 | -66.3755 | -33.6245 | -3.93013 | -5.67686 |
| NS | 0 | -10.6061 | -7.07071 | 3.535354 | 6.060606 | -1.0101 | -3.0303 |
|  | 0 | -0.69444 | -1.73611 | 1.736111 | 4.166667 | -3.125 | -1.73611 |
|  | 0 | -3.83481 | -6.19469 | -0.58997 | 5.309735 | 10.91445 | -0.88496 |

| **vF 4** | baseline | 5 | 10 | 20 | 30 | 60 | 90 (min) |
| --- | --- | --- | --- | --- | --- | --- | --- |
| Levobupivacaine (0.05%) | 0 | 7.692308 | -2.30769 | -60 | -34.6154 | -30 | -13.0769 |
|  | 0 | -23.9316 | 6.837607 | 0 | 29.05983 | 22.22222 | 62.39316 |
|  | 0 | -58.3893 | -63.0872 | -51.0067 | -22.8188 | -25.5034 | -25.5034 |
|  | 0 | -26.5089 | -43.1953 | -55.9763 | -20.4734 | -24.0237 | -14.4379 |
|  | 0 | 6.338028 | 9.15493 | -0.70423 | -18.3099 | -11.2676 | -3.52113 |
| Bupivacaine (0.05%) | 0 | -84.127 | -93.6508 | -77.7778 | -88.0952 | -32.5397 | 11.90476 |
|  | 0 | -73.3333 | -85.4545 | -76.3636 | -38.1818 | -27.2727 | 3.636364 |
|  | 0 | -0.68966 | -28.2759 | -81.3793 | -57.931 | -31.0345 | -4.82759 |
|  | 0 | -66.1972 | -66.9014 | -72.5352 | -57.7465 | -4.92958 | -19.0141 |
|  | 0 | -61.157 | -65.7025 | -72.7273 | -45.0413 | -17.3554 | -22.314 |
| NS | 0 | -10.0559 | 0 | -3.35196 | 0 | -6.70391 | 2.793296 |
|  | 0 | 6.666667 | 8.205128 | -1.53846 | 4.102564 | 2.564103 | 6.153846 |
|  | 0 | -10.6061 | -7.07071 | -2.52525 | 6.060606 | -1.0101 | -3.0303 |
