# Supplementary material for: Differences in sensory nerve block between levobupivacaine and bupivacaine at low concentrations in humans and animals
Source: PLoS One. 2025 Feb 10;20(2):e0306591. doi: 10.1371/journal.pone.0306591 (PMC11809910; doi:10.1371/journal.pone.0306591)
Supplement: Attachment — Submitted filename: Response to Reviewers(3).docx [file pone.0306591.s004.docx]

We would like to thank the academic editor and reviewers for carefully reading our manuscript and for the insightful comments. The comments led to an improvement of the work. Detailed responses to the reviewers are shown below. The comments from the editor and reviewers are shown in black and our replies are shown in red. In this revised version, changes to our manuscript within the document were all highlighted by using red-colored text (**with underlining**) in the file named “**Revised Manuscript with Track Changes**”.

Reply to academic editor

Please review your reference list to ensure that it is complete and correct. If you have cited papers that have been retracted, please include the rationale for doing so in the manuscript text, or remove these references and replace them with relevant current references. Any changes to the reference list should be mentioned in the rebuttal letter that accompanies your revised manuscript. If you need to cite a retracted article, indicate the article’s retracted status in the References list and also include a citation and full reference for the retraction notice.

On January 12, 2025, we re-evaluated all references 1 to 32 of our manuscript (PONE-D-24-22812) using the literature search system “Medline” and we confirmed that there were no retracted papers. Therefore, no deletions or changes to the references are necessary.

Reply to Reviewer #1

Reviewer #1: Minor revisions:

Line 212: State the statistical testing method which achieves 80% power. Examples of testing methods are t-test, ANOVA, and Fisher's Exact test etc.

Thank you for pointing this out.

We used the t-test for our 80% power statistic. Therefore, we have rewritten the text of Line 212 as follows.

Line 212: We used G-Power 3.1 to determine the sample size, with a type I error rate (α) of 0.05, power value of 80% (1 − β = 0.80), an effect size of 2, and two-sided t-test.

Before the start of the study, we anticipated that the data would be normally distributed and considered using a t-test for statistical analysis. However, since the data did not exhibit normality, we conducted statistical analysis using the Mann-Whitney U test. With a type I error rate of 0.05, a power value of 80% (1 − β = 0.80), and an effect size of 2, the required sample size for the Mann-Whitney U test was calculated to be n = 6 using G-Power 3.1. In any case, we believe that this study was conducted with an appropriate sample size.
